# What are farmers really planting? Measuring the presence and effectiveness of Bt cotton in Pakistan

**DOI:** 10.1371/journal.pone.0176592

**Published:** 2017-05-04

**Authors:** David J. Spielman, Fatima Zaidi, Patricia Zambrano, Asif Ali Khan, Shaukat Ali, H. Masooma Naseer Cheema, Hina Nazli, Rao Sohail Ahmad Khan, Arshad Iqbal, Muhammad Amir Zia, Ghulam Muhammad Ali

**Affiliations:** 1 Environment and Production Technology Division, International Food Policy Research Institute, Washington, DC, United States of America; 2 Muhammad Nawaz Shareef University of Agriculture, Multan, Pakistan; 3 Office of Research, Innovation and Commercialization, University of Agriculture Faisalabad, Faisalabad, Pakistan; 4 National Institute for Genomics and Advanced Biotechnology, Pakistan Agricultural Research Council, Islamabad, Pakistan; 5 Department of Plant Breeding and Genetics, University of Agriculture Faisalabad, Faisalabad, Pakistan; 6 Pakistan Strategy Support Program, International Food Policy Research Institute, Islamabad, Pakistan; 7 Centre of Agricultural Biochemistry and Biotechnology, University of Agriculture Faisalabad, Faisalabad, Pakistan; Pennsylvania State University, UNITED STATES

## Abstract

Genetically modified, insect-resistant *Bacillus thuringiensis* (Bt) cotton is cultivated extensively in Pakistan. Past studies, however, have raised concerns about the prevalence of Bt cotton varieties possessing weak or nonperforming insect-resistance traits conferred by the *cry* gene. We examine this issue using data drawn from a representative sample of cotton-growing households that were surveyed in six agroclimatic zones spanning 28 districts in Pakistan in 2013, as well as measurements of Cry protein levels in cotton tissue samples collected from the sampled households’ main fields. The resultant dataset combines information from 593 sampled households with corresponding plant tissue diagnostics from 70 days after sowing, as well as information from 589 sampled households with corresponding diagnostics from 120 days after sowing. Our analysis indicates that 11 percent of farmers believed they were cultivating Bt cotton when, in fact, the Cry toxin was not present in the tested tissue at 70 days after sowing (i.e., a Type I error). The analysis further indicates that 5 percent of farmers believed they were cultivating non-Bt cotton when, in fact, the Cry toxin was present in the tested tissue (i.e., a Type II error). In addition, 17 percent of all sampled farmers were uncertain whether or not they were cultivating Bt cotton. Overall, 33 percent of farmers either did not know or were mistaken in their beliefs about the presence of the *cry* gene in the cotton they cultivated. Results also indicate that toxic protein levels in the plant tissue samples occurred below threshold levels for lethality in a significant percentage of cases, although these measurements may also be affected by factors related to tissue sample collection, handling, storage, and testing procedures. Nonetheless, results strongly suggest wide variability both in farmers’ beliefs and in gene expression. Such variability has implications for policy and regulation in Pakistan’s transgenic cotton seed market.

## Introduction

Genetically modified, insect-resistant *Bacillus thuringiensis* (Bt) cotton was officially released in Pakistan in 2010 when the National Biosafety Committee (NBC), operating under the auspices of the Pakistan Environmental Protection Agency (EPA), approved commercialization of nine Bt cotton varieties containing first-generation *cry* genes that confer resistance to Lepidopteran pests. However, extensive evidence indicates that some farmers had access to Bt cotton varieties several years earlier [1,2,3]. Evidence also suggests that both the earlier unapproved varieties and the later approved varieties express the introgressed trait in an inconsistent and possibly ineffective manner [4,5]. A weak, ineffective regulatory system governing the commercialization of transgenic events, the release of new varieties, and the distribution of seed—alongside of the inherent difficulty farmers face in evaluating the quality of a given trait, variety, or seed upon visual inspection at the time of purchase—may have collectively contributed to the proliferation of spurious or low-quality Bt cotton in Pakistan’s cotton-growing areas [6–9].

There are several potential risks associated with low-quality Bt cotton and, specifically, low (or absent) expression levels of the *cry* gene that leads to production of a soluble protein toxic to Lepidopteran pests. First, weak expression can reduce the damage abatement characteristics of the *cry* gene, thereby limiting the associated benefits of production cost reductions, yield improvements, or both. Second, weak expression can cause farmers to revert to high levels of pesticide application to control the pests that the *cry* gene is otherwise meant to control, thereby eliminating the cost and environmental benefits associated with a reduction in the number of insecticide applications. Third, weak expression can contribute to the emergence of resistance in the target pest population via natural selection.

A systematic exploration of this problem requires close consideration of two related measurement problems. First is the measurement of the biological aspects of the problem—the presence and expression levels of the Bt protein produced by the cotton plant. Second is the measurement of the social and economic aspects—the farmers’ own understanding of whether that Bt gene is embodied in the cotton variety they purchased and sowed, as well as the individual-, household-, and farm-level factors that influence that understanding. This study explores these two measurement issues to understand whether the presence and expression levels of the Bt gene in cotton are sufficient to provide effective resistance to targeted pests in Pakistan, as well as whether there is variability in the types of farmers who have access to more effective Bt cotton varieties.

## Background and context

While different *cry* genes from the Bt bacterium have been used to confer the insect-resistance trait in cotton (including *cry1Ac*, *cry1Ab*, *cry2Ab* and *cry1F*), most Bt cotton varieties in Pakistan rely on the *cry1Ac* gene from the MON-531 event developed by Monsanto that is introgressed into locally improved germplasm [6]. Monsanto, a U.S.-based multinational company and global leader in genetically modified seeds and traits, first commercialized its *cry1Ac* gene in the United States in 1996, marketing it under the Bollgard trademark. At the time the *Cry1Ac* gene was introduced into Pakistani cotton varieties, Monsanto did not hold a patent on the gene in Pakistan.

The gene is effective in controlling certain types of Lepidopteran pests such as American bollworm (*Helicoverpa zea*), old world bollworm (*Helicoverpa armigera*), pink bollworm (*Pectinophora gossypiella*), spiny bollworm (*Earias spp*.), and tobacco budworm (*Heliothis virescens*). It is less effective, however, against cotton leafworm (*Spodoptera litura*) and fall armyworm (*Spodoptera frugiperda*), although resistance has emerged in some countries (e.g., pink bollworm [10]). By reducing losses from bollworm damage, Bt cotton has become an important damage abatement technology in major cotton-producing countries worldwide.

This is also the case in Pakistan. While Bt cotton does not provide resistance to common sucking pests such as thrips, jassids, whiteflies, and mealybugs, or diseases such as cotton leaf curl virus, it was effective in reducing bollworm damage, lowering pesticide costs, and curtailing some of the environmental consequences of pesticide use, even despite higher seed costs for Bt cotton [11–14, 2].

The issue of Bt protein expression has been explored previously in Pakistan. Ali et al. [5] conducted a survey in 10 districts in Sindh and 11 districts in Punjab during the 2007–08 cotton season using immunostrip tests, and found that 10 percent of the samples taken in Punjab and 19 percent in Sindh tested non-positive for the *cry1Ac* gene. Of those samples that tested positive for the *cry1Ac* gene, only 41 percent in Sindh and 36 percent in Punjab were found to contain Bt protein concentrations sufficiently lethal to cause mortality in the targeted pest, although a precise measure was not indicated in the study [5]. The remainder exhibited either medium or low levels of toxin expression. Ali et al. concluded that such low levels of toxin expression may be attributable to seed mixing (adulteration), poor breeding methods that fail to recover the gene of interest in the recurrent parent, or improper genetic backgrounds in the host. In 2011, Ali et al. [4] conducted a similar study, using immunostrip tests to analyze samples of seeds sold in the market as Bt cotton. After planting and cultivating these seeds, the authors tested the plants for *cry* gene expression. Results from these tests showed that 30 percent (14 out 46) of the varieties tested non-positive for any *cry* gene. Moreover, findings from these studies are generally consistent with the larger literature examining factors that influence variable Cry protein levels in Bt cotton, including genetic background, climatic conditions, agronomic practices, abiotic stresses, plant growth stage, and plant tissue type [15–20].

Cry protein levels can also be influenced by factors related to the techniques employed in genetic modification and breeding, including the efficacy of the gene being deployed, the genetic background into which it is introgressed, the techniques used to introgress the gene, and the practices used in breeding and seed multiplication. Poor quality backcrossing, gene segregation in F1 generations, heterozygosity, variation in nucleotide sequences, the type of promoters used, and the insertion site in the host DNA can all affect gene expression and thus Cry protein levels [21, 22, 23]. Factors such as seed source, seed package adulteration, moisture, and contaminants are also found to affect Cry protein levels [15,24].

## Materials and methods

Because the objectives of this study require both biophysical data and household data, it was not feasible to use standard procedures in which Bt cotton varieties are evaluated under controlled experimental conditions in a greenhouse. Instead, we collected and measured cotton tissue samples in situ using a strict collection protocol, and then tested the samples for Bt presence and expression levels. We also conducted a household survey designed to collect data on the cotton variety sown by the household, whether (and if so, when) the household adopted Bt cotton, the farming practices employed, individual farmer attributes, and characteristics of the household and farm that can be used to explain variability in the presence and performance of the Bt trait where present. This approach differs significantly from prior studies that combine socioeconomic and biophysical data but rely on controlled experimental conditions to generate the latter type of data [25]. Our approach allows for the analysis of multiple issues associated with Bt cotton cultivation in Pakistan, including smallholder farmers’ input-use decisions and crop management practices that influence cotton yields, production, and the abatement of damage caused by targeted pests (see, e.g., Ma et al. [11]). However, the approach also poses certain logistical challenges, as described in further detail below.

Data were collected with approval from the Institutional Review Board (IRB) of the International Food Policy Research Institute (IRB no. 00007490; FWA no. 00005121) and are available in the public domain at https://dataverse.harvard.edu/dataverse/IFPRI. Participants in this study were read the informed consent form and asked to provide their informed consent verbally. This procedure was pursued due to high rates of illiteracy in the study area, particularly among elderly and women respondents. Enumerators recorded the verbal affirmation or refusal of consent on the first page of the questionnaire. Where consent was refused, the interview was discontinued immediately. This procedure received the approval of the aforementioned IRB.

The primary unit of analysis in this study is cotton-growing households in all agroclimatic zones where cotton is cultivated. The sampling frame for the household survey was designed as follows: First, Pakistan’s main cotton-growing areas were stratified into six agroecological zones (AEZs) that account for more than 99 percent of the cotton cultivated in Pakistan [26, 27]. Second, 52 *mauzas* (revenue villages) were randomly selected from these six AEZs based on probabilities proportional to population size (PPS), so that 40 *mauzas* were selected in Punjab and 12 in Sindh. Third, 14 households were randomly selected in each village based on equal probabilities. The resulting sample of 728 households is representative of the population in Pakistan’s main cotton-growing agroclimatic zones.

A series of household interviews were conducted during and after the 2013 *kharif* (monsoon) season with the head of each household or the individual most knowledgeable about cotton cultivation. The interviews were based on a structured questionnaire designed to obtain data on individual attributes and household characteristics, as well as crop- and plot-level data on the cotton varieties sown, production inputs (seed, fertilizer, agrochemicals, and labor), and harvest. Data were collected from respondents on both their 2012 and 2013 *kharif* cotton crop. The surveys were designed and implemented by the Pakistan Strategy Support Program, an initiative led by the International Food Policy Research Institute in collaboration with Innovative Development Strategies (S1 Tables) [11].

The biophysical component of this study unfolded as follows (see S1 Appendix for details). In each of the sampled households, the survey respondent was asked to identify the household’s main cotton plot. The biophysical survey team collected leaf and boll samples from five randomly selected plants in that main plot. Tissue samples were collected twice over the growing season—once at approximately 70 days after sowing (DAS) and a second time at approximately 120 DAS. The first collection round at 70 DAS took place between June and August 2013; the second round at 120 DAS took place between August and October 2013. Collection at 70 and 120 DAS are approximations because of variability in planting dates across the sample made collection on precise dates logistically challenging. Nonetheless, collections were successfully conducted within a week of the 70 and 120 DAS point because of close correlations in planting dates among households within the same AEZs and districts.

On each plot, each of the five plants was tagged during the first collection round (70 DAS) so that they could be identified for collection at 120 DAS. For each plant, two leaves of similar size, color, and age were collected from the upper third of the plant. The leaf samples from each of the five plants were then placed in pre-labeled polythene zip-lock bags and immediately stored in dry ice containers for transportation to the testing laboratory sites. A similar procedure was carried out for bolls, with two bolls collected from each of the same sampled plants, placed on dry ice in zip-lock bags, and labeled accordingly. Specimen collection and laboratory testing (described in detail below) were conducted in Punjab by the University of Agriculture, Faisalabad (UAF), as well as in Sindh by the National Institute of Genomics and Biotechnology (NIGAB), Islamabad (S1 Appendix).

Since the biophysical and household surveys were conducted by different teams, within different time periods, and subject to the availability of plant tissue at the same stage of growth, the number of households included in the biophysical survey differs slightly from that of the household survey. Of the original 728 households sampled, 46 chose not to grow cotton during the 2013 *kharif* season, 70 lost their crops to flood or other natural disasters, 4 migrated, 8 dropped out in the second or third round of surveys, and 29 did not participate in the corresponding biophysical component of the study. A total of 593 main cotton plots were identified during the first round of tissue sample collection at 70 DAS, and a total of 589 plots were identified during the second round at 120 DAS (Table 1). Summary statistics for those who remained in the sample and those who did not suggest that the two groups of farmers are not significantly different, suggesting further that the sample retained decent coverage of the populations and agroclimatic zones of interest [11].

**Table 1:** Number of household and plot samples, 2013.

*Household/plot sample*	70 DAS			120 DAS		
	Punjab	Sindh	Total	Punjab	Sindh	Total
Planned	560	168	728	560	168	728
Tissue collected	461	132	593	457	132	589
Tissue not collected due to…						
Non-cultivation	86	28	114	103	28	131
Natural disaster		8	8	—	8	8
Out migration	—	—	—	—	—	—
Misplacement	5	—	5	—	—	—
Other reasons	8	—	8	—	—	—

Source: Authors

Laboratory tests conducted on plant tissue samples were administered as follows: UAF and NIGAB used identical equipment to test for both (1) the presence of *cry* genes, using lateral flow strip assays commercially marketed as QuickStix Combo Kits and manufactured by EnviroLogix Inc., and (2) the expression levels of Bt protein, using ELISA kits commercially sold by the same manufacturer as QualiPlate Combo Kit for Cry1Ac and Cry2Ab. The methods and materials used were generally consistent with prior studies designed around the detection of Cry proteins [28–31, 5] and the quantification of Cry protein levels [29, 30, 32, 33, 20, 17, 5]. Both tests are qualitative in nature, providing laboratory detection of a range of Cry proteins in cotton leaf or seed samples. The ELISA kit is also sensitive enough to provide quantitative applications with appropriate procedures and calibrations. The procedures and calibrations (detailed in S1 Appendix) followed the manufacturer’s guidelines and recommendations, and are similar to those described in Liu et al. [34]. Several logistical problems were encountered in the field that limited the ability to adhere to a consistent procedure across Punjab and Sindh. These problems were associated with differences in geographic distances between sample sites and labs, transportation constraints, electrical power outages, and other infrastructural constraints beyond the control of UAF and NIGAB. As a result, the samples collected from farmers’ fields were ultimately handled differently between Punjab and Sindh. In Punjab, the tissue samples were transported to UAF’s laboratories under -20°C storage conditions using dry ice and maintained in a cold storage refrigerator at the same temperature for 7–15 days until tests were performed. In Sindh, the strip tests were performed on site immediately after collection, while the ELISA tests were performed within 24 hours of collection at one of two nearby university laboratories using the fresh tissue samples transported on cold gelpacks at 4–10°C.

As prescribed in the protocol, both diagnostic tests were to be performed only on two of the five leaf samples and two of the five boll samples collected from each household’s main cotton plot. The remaining three samples of both bolls and leaves were to be stored at -80°C for follow-up tests and third-party evaluation. This procedure was followed in Punjab. In Sindh, however, strip tests were performed consecutively on each of the five tissue samples collected from the field until two positives were attained, and subsequently only positive results were subject to the ELISA test. In total, 9,153 diagnostic tests were conducted on the two types of plant tissue collected.

There were also differences in the way the samples were weighed for testing as well as for the subsequent calculation of Bt protein expression levels. In Punjab, the actual weight was measured for each tissue sample, while in Sindh a constant sample weight of 20 mg was used in calculations. There were also minor procedural differences between testing in Punjab and Sindh, such as the choice of dilution factor (1:11 in Punjab and 1:9 in Sindh). Lastly, the procedures prescribed that the sample tissue be stored at -80°C, but due to continuous power outages and load shedding, this temperature may not have been maintained throughout the entire period in which this study was conducted. Thus our analysis and interpretation of the results is constrained due to the different procedures and conditions under which tissue samples were collected, transported, stored, and tested.

## Results

### Adoption rates based on farmer self-reporting and strip test results

Self-reported farmer data collected in the household survey indicate that the earliest reported year in which Bt cotton was cultivated was 2003, although this was reported by just two (0.3 percent) farmers in the sample. About 51 percent of sampled farmers reported that they had adopted Bt cotton during the three years preceding the survey (2010–2012). By 2013, 85 percent of sampled farmers reported that they had planted Bt cotton. Table 2 shows that Bt cotton adoption accelerated in 2010, which coincides with the year in which Pakistan first approved the commercialization of Bt cotton varieties. Note that these reported adoption rates obscure significant provincial heterogeneity; although adoption rates in Punjab had reached 98 percent by 2013, rates in Sindh lagged behind at 42 percent.

**Table 2:** Cumulative adoption rates for Bt cotton, by year and province.

Year	Respondents reporting to have adopted Bt cotton (%, cumulative)		
	Punjab	Sindh	Total
2003	0	1	0
2004	0	1	0
2005	1	4	2
2006	5	4	5
2007	9	8	9
2008	20	14	19
2009	36	18	32
2010	65	25	56
2011	86	32	73
2012	96	40	83
2013	98	42	85

Source: Authors.

The results from the strip tests generate slightly different findings. We assume that at least one positive strip test result indicating that the sampled tissue contains the Bt gene further indicates that the plot is sown with Bt cotton and the farmer has adopted Bt cotton. Based on these assumptions, findings suggest an overall adoption rate of 80 percent as of 2013–82 percent in Punjab and 71 percent in Sindh. Thus self-reported adoption rates may be overestimated by 4 percentage points overall and by 16 percentage points in Punjab, but underestimated by 29 percentage points in Sindh. This is an indication of the possible discrepancies in farmer perceptions (Table 3).

**Table 3:** Results from strip tests for the presence of the Bt gene at 70 days after sowing, by province, 2013.

Results from tests of leaf samples 1 and 2	Punjab		Sindh		Total	
	*#*	*%*	*#*	*%*	*#*	*%*
Both negative	82	17.8	38	28.8	120	20.2
One positive/one negative	145	31.5	23	17.4	168	28.3
Both positive	234	50.8	71	53.8	305	51.4
Total	461	100	132	100	593	100

Source: Authors.

Note: The results reported here are based on strip tests of leaf tissue collected at 70 DAS. A positive result indicates that at least one strip test of a tissue sample from a single plant tested positive for the *cry* gene.

#### Type I and Type II errors

The findings above open the door to an analysis of discrepancies between farmer perceptions and diagnostic test results for Bt cotton. Here, we explore inconsistencies between what farmers believe about the cotton they plant—whether it is Bt cotton or not—and the what the results of the diagnostic tests reveal about plant tissue collected from the farmers’ main cotton plot. Drawing on Maredia and Reyes [35], we describe these cases as Type I and Type II errors to provide a more intuitive analysis of the data. We denote as Type I errors cases in which a farmer’s belief is a false positive—that is, a farmer reported planting Bt cotton, but the corresponding strip test results from the farmer’s main plot indicate that Bt cotton was not planted. We denote as Type II errors cases in which a farmer’s belief is a false negative—that is, the farmer reported planting non-Bt cotton, but the corresponding strip test results from the farmer’s main plot indicate that Bt cotton was planted. We explore the prevalence of these errors in our sample below.

Table 4 reports findings from Punjab in the upper panel and Sindh in the lower panel. Keep in mind that the difference in collection, transport, storage, and testing procedures between the two provinces necessitates caution in directly comparing provincial results. In Punjab, the procedure was to test only two leaf tissue samples taken from the plot, regardless of the result obtained. In Sindh, the procedure was to consecutively test all five tissue samples collected from the plot until two positive results were acquired, if possible. Assuming that at least one leaf sample returning a positive test result indicates the presence of the Bt gene, then we can determine the prevalence of Type I and II errors among farmers in the sample based on the findings reported in Table 4.

**Table 4:** Comparison of farmers’ perceptions and strip test results for presence of the Bt gene, 70 DAS, 2013.

**Farmers' belief**	**Punjab: Strip tests results**													
	**Both negative**			**One positive, one negative**				**Both positive**			**Total**		**Total no. and % of plots found to contain Bt cotton**	
	**#**		**%**	**#**		**%**		**#**	**%**		**#**	**%**	**#**	**%**
Bt	61		17.1	116		32.5		180	50.4		357	100.0	296	82.9
Non-Bt	13		43.3	8		26.7		9	30.0		30	100.0	17	56.7
Don't know	6		11.8	15		29.4		30	58.8		51	100.0	45	88.2
No response	2		8.7	6		26.1		15	65.2		23	100.0	21	91.3
Total	82		17.8	145		31.5		234	50.8		461	100.0	379	82.2
**Farmers' belief**	**Sindh: Strip tests results**													
	**All negative**		**1 out of 5 tests positive**				**2 out of 3 or more tests positive**		**2 out of 2 tests positive**		**Total**		**Total no. and % of plots found to contain Bt cotton**	
	**#**	**%**	**#**		**%**		**#**	**%**	**#**	**%**	**#**	**%**	**#**	**%**
Bt	1	2.0	1		2.0		14	28.0	34	68.0	50	100.0	49	98.0
Non-Bt	14	50.0	0		0.0		4	14.3	10	35.7	28	100.0	14	50.0
Don't know	17	33.3	1		2.0		7	13.7	26	51.0	51	100.0	34	66.7
No response	1	33.3	1		33.3		0	0.0	1	33.3	3	100.0	2	66.7
Total	33	25.0	3		2.3		25	18.9	71	53.8	132	100.0	99	75.0

Source: Authors

Overall, the strip test results indicate that 82 percent of the plots sampled in Punjab at 70 DAS were planted with Bt cotton. With 77 percent of farmers believing they were cultivating Bt cotton, there is a seemingly strong level of correspondence between farmers’ perceptions and strip test results in Punjab. However, the extent of non-correspondence between perceptions and strip test results warrants closer scrutiny. In Punjab, Type I errors occurred in 17 percent of cases where farmers reported that they *did* plant Bt cotton, while Type II errors occurred in 57 percent of cases where farmers reported that they *did not* plant Bt cotton. In short, Type I and Type II errors account for 17 percent of all cases described for Punjab in Table 4. Findings also indicate that 11 percent of farmers sampled in Punjab reported that they did not know if they had planted Bt cotton. Among these farmers, 88 percent were *unknowingly* cultivating Bt cotton, while the remaining 12 percent were *unknowingly* cultivating non-Bt cotton. Taken together, 27 percent of farmers either committed Type I or Type II errors, or were simply unaware of whether they were cultivating Bt cotton at all.

In Sindh, strip test results suggest that 75 percent of the sampled plots were planted with Bt cotton. Yet just 38 percent of farmers actually believed they were cultivating Bt cotton. Findings from Sindh further suggest the following: First, 98 percent of the cases in which farmers believed that they *did* plant Bt cotton were consistent with strip test results that were *positive* for the Bt gene in at least one test. In other words, Type I errors occurred in just 2 percent of cases where farmers reported that they had planted Bt cotton. Second, just 50 percent of the cases in which farmers believed that they *did not* plant Bt varieties were consistent with strip test results that were negative for the Bt gene in at least one test. In other words, Type II errors occurred in 50 percent of cases where farmers reported that they had not planted Bt cotton. Overall, Type I and Type II errors account for 11 percent of all cases from Sindh.

In addition to this, 39 percent of farmers sampled in Sindh reported that they did not know if they had planted Bt cotton. Among these farmers, 67 percent were *unknowingly* cultivating Bt cotton, while the remaining 33 percent were *unknowingly* cultivating non-Bt cotton. In total, 37 percent of the sampled farmers committed Type I or Type II errors, or were simply unaware of whether they were cultivating Bt cotton at all.

Combining the data for Sindh and Punjab, overall findings suggest that while self-reported adoption rates were 85 percent in 2013, strip test results suggest that Type I and Type II errors account for 16 percent of all cases, with Type I errors accounting for 11 percent and Type II errors accounting for 5 percent. Cases in which farmers did not know whether they were cultivating Bt cotton account for an additional 17 percent of all cases. Overall, 33 percent of farmers either did not know or were mistaken in their beliefs about the presence of the Bt gene in the cotton they cultivated.

But just as farmers’ self-reported adoption rates may be inaccurate, there is also a possibility (though somewhat more remote) that the strip test results are inaccurate. There are several technical considerations that might limit the reliability of strip tests. They include the possibility of false positive results caused by temperature, humidity, cross-contamination by other samples, or other factors [36]. That said, the results described above are in agreement with tests conducted on tissue samples collected at 120 DAS in both Punjab and Sindh, as well as with ELISA results (described below) that were used simply to detect the presence or absence of the Bt protein (S2 Appendix).

Finally, strip test results from both provinces did not indicate the presence of the Cry2Ab proteins, which includes Monsanto’s Bollgard II containing the MON-15985 event. This suggests that the only transgenic event introgressed into Pakistani cotton varieties is *cry1Ac*.

### Efficacy of Bt gene expression

Next, we analyze results from the ELISA tests conducted to measure Bt expression levels and estimate the efficacy of Bt gene expression. Unlike the strip tests, which can be conducted in the field, ELISA tests require meticulous laboratory handling, careful adherence to procedures prescribed in the kit, and a variety of calculations to generate valid measurements. ELISA results are sensitive to laboratory conditions (temperature, airflow, and other factors), tissue weight, storage temperature, and tissue handling. For this reason, we suggest caution in the interpretation of results with respect to gene expression and Bt cotton performance.

Table 5 summarizes the results of all ELISA tests for Sindh and Punjab and provides statistics on the toxin expression levels for leaf and boll tissue collected from farmers’ main plots at 70 and 120 DAS. These summary statistics are based on all positive ELISA results (all observations that were non-missing and positive), irrespective of strip test results.

**Table 5:** ELISA results for Bt-positive plant tissue samples, Punjab and Sindh (µg/g).

Province, tissue, date of collection	Observations (no.)	Mean (μg/g)	Std. Dev.	Min (μg/g)	Max (μg/g)
*Punjab*					
Leaf, 70 DAS	922	0.98	0.81	0.00	5.18
Leaf, 120 DAS	909	0.70	0.64	0.00	3.75
Boll, 70 DAS	815	0.59	0.47	0.00	2.88
Boll, 120 DAS	851	0.53	0.51	0.00	4.07
*Sindh*					
Leaf, 70 DAS	197	2.43	1.97	0.01	8.31
Leaf, 120 DAS	197	1.85	1.34	0.04	5.53
Boll, 70 DAS	170	0.67	0.81	0.01	4.69
Boll, 120 DAS	182	0.63	0.52	0.01	2.85

Source: Authors

Note: Minimum ELISA results of 0.00 μg/g indicate that the sample tested positive according to the strip tests but did not contain a level of toxin detectable by ELISA.

Findings indicate a statistically significant difference in both the means and distributions of Bt protein expression between Punjab and Sindh. While the highest mean ELISA reading (with standard errors in parentheses) for Bt-positive tissues tested in Punjab is 0.98 μg/g (± 0.027), the equivalent mean in Sindh is more than double at 2.43 μg/g (± 0.140). This is likely attributable to the differences in the procedures followed and the prevailing field conditions in Punjab and Sindh, making comparisons across provinces difficult.

Concerns about in situ tissue sample collection under difficult field conditions and the sensitivity of ELISA results prompted the analysis of tissue samples taken from the same plant. Specifically, we compare the first sample of a particular tissue type against the second sample of the same tissue type, collected from the same plant at the same time. We include in this analysis only observations for which the strip test was positive, and we dropped observations for which we did not have paired observations, either because the expression levels were below detectable levels in the ELISA test or, in the case of bolls, because bolls had not yet formed at the time of collection. Results indicate that within-plant levels of Bt protein expression are not different (Table 6). These findings are consistent for both leaves and bolls, as well as for both Punjab and Sindh.

**Table 6:** Within-tissue comparisons of Bt protein expression levels, 2013.

Province, time of collection, and tissue sample tested	Mean level of Bt gene expression level for		No. of (paired) observation	p-value for difference
	tissue sample 1	tissue sample 2		
*Punjab*				
70 DAS, leaf	0.98 (0.04)	0.98 (0.04)	461	0.91
120 DAS, leaf	0.73 (0.03)	0.67 (0.03)	457	0.10
70 DAS, boll	0.61 (0.02)	0.56 (0.02)	409	0.06
120 DAS, boll	0.54 (0.02)	0.51 (0.03)	415	0.25
*Sindh*				
70 DAS, leaf	1.82 (0.18)	1.87 (0.18)	129	0.75
120 DAS, leaf	1.38 (0.12)	1.40 (0.13)	129	0.75
70 DAS, boll 120 DAS, boll	0.52 (0.08) 0.50 (0.05)	0.46 (0.07) 0.44 (0.05)	113 117	0.50 0.18

Source: Authors

Note: Standard errors in parentheses.

We use these results to estimate the efficacy of Bt gene expression in the sampled tissues. A positive indicator of Bt gene expression, whether based on strip test or ELISA test results, does not necessarily guarantee that the Bt cotton plant will effectively control the targeted pest. To determine if an insecticide is likely to control the targeted insect population, commonly accepted practice is to establish a threshold based on controlled experiments in which plant tissue is fed to targeted pests and their mortality rates are measured. To establish this threshold for our sample, we rely on prior studies of *H*. *armigera* that set lethal Cry protein levels—the lethal dose at which the targeted insects die after ingesting the Bt plant tissue—within a range between 0.45 μg/g and 1.90 μg/g [15, 17].

We examine our samples 0.6 μg/g, 1.4 μg/g and 1.6 μg/g, all of which fall within the range of levels set by these prior studies. These threshold levels were then used to both evaluate the lethality of leaf samples collected at 70 DAS and to revise the classification of whether farmers were planting Bt or non-Bt cotton in their main plots. The classification categorizes leaf tissue samples collected at 70 DAS into three categories. The “Bt” category denotes levels of the Cry protein above the thresholds in both samples tested, and the “non-Bt” category denotes levels below these thresholds. The inconclusive category denotes instances where the levels of the Cry protein for one leaf sample equal or exceed the threshold but fall below the threshold for the other leaf sample.

Results reported in Table 7 indicate that at a threshold of 1.6 μg/g, just 10 percent of plant tissue samples contained lethal levels of the Cry protein. At a threshold of 1.4 μg/g, the figure increases to 19 percent and, at 0.6 μg/g, to 62 percent. But, as noted earlier, we suggest caution in the interpretation of results with respect to Bt expression levels and efficacy.

**Table 7:** Classification of Bt cotton leaf tissue samples collected at 70 DAS based on threshold levels of Cry protein, by province, 2013.

Bioassay classification	at 0.6 ug/g		at 1.4 ug/g		at 1.6 ug/g	
	#	%	#	%	#	%
*All*						
Bt	367	61.9	114	19.2	85	9.7
Non-Bt	3	0.5	346	58.3	390	72
Inconclusive	223	37.6	133	22.4	118	18.2
Total	593	100	593	100	593	100
*Punjab*						
Bt	290	62.9	74	16.1	46	10.0
Non-Bt	-	-	290	62.9	329	71.4
Inconclusive	171	37.1	97	21.0	86	18.7
Total	461	100	461	100	461	100
*Sindh*						
Bt	77	58.3	40	30.3	39	29.5
Non-bt	3	2.3	24	18.2	29	22.0
Inconclusive	52	39.4	68	51.5	64	48.5
Total	132	100	132	100	132	100

Source: Authors

## Exploring the correlates of (mistaken) beliefs

Why might farmers’ perceptions conflict with test results? First, it is possible that the sampled farmers may have unknowingly planted a mix of Bt and non-Bt cotton on their plot or on their farm as a whole—perhaps as a result of intentional or unintentional mixing of seed by seed retailers. Second, it is possible that the sampled farmers knowingly planted a mix as part of an intentional strategy—for example, to manage risk by sowing multiple varieties across or within plots or to manage the emergence of pest resistance by establishing an informal refugia within the farm. Since the strip test results reported here are based only on tissue samples taken from plants in each farmer’s *main* cotton plot, it is impossible to use these data to measure the intensity at which Bt cotton was cultivated on an entire farm or the extent to which seed mixing, variety or trait diversification, or refugia strategies might explain the presence of both Bt and non-Bt cotton. However, the data do suggest that intentional strategies in which farmers plant multiple varieties are relatively rare, thereby ruling out risk management or refugia strategies as an explanation of the discrepancies between farmers’ reported cultivation of Bt cotton in their main plots and our test results. Among the 15 percent of sampled farmers who cultivated two or more plots in the 2013 *kharif* season, just 6 percent planted two or more varieties on their main plot, and just 11 percent planted two or more varieties on their entire farm. It is unlikely, however, that farmers were cultivating Bt and non-Bt cotton plots separately—whether unintentionally or strategically—in part, because only 6 percent of sampled households indicated that they had heard about refugia recommendations and none indicated following them, effectively ruling out one situation that might explain the systematic planting of non-Bt cotton alongside Bt cotton at a farm or plot level.

Third, it is possible that farmers pay little attention to, or place little value on, the Bt trait and thus chose to commit Type I or Type II errors with little consequence. This could be the case given a significant reduction in bollworm pressure in Pakistan that would have followed from rapid and widespread adoption of Bt cotton between 2005 and 2010, and may be reflected in the negligible (and in our sample, statistically insignificant) price difference between Bt and non-Bt cotton seed. In short, there may be evidence to suggest that the Bt trait simply does not matter to farmers anymore. Running counter to this conclusion, however, is the fact that fully 34 percent of sampled farmers in 2013 still identified bollworms as the most significant stress facing cotton production. Moreover, recent reports indicate that Bt resistance to pink bollworm has emerged in Pakistan as it did in India, suggesting that farmers may have had reason to retain their concerns about the threats posed by bollworms [37, 38].

Having now established that a significant share of households is mistaken in their beliefs about whether they cultivated Bt cotton, we turn to the question of what factors might drive such mistaken decisions. We explore this in the context of two discrete choice models, although we recognize that the mistaken beliefs are not “choices” per se. For this reason, these models examine the correlates that might help characterize the types of households that are more likely to hold mistaken beliefs.

The first model uses a logit estimation to examine correlates of the accuracy of a household’s beliefs—that is, whether beliefs are consistent with the test results, or:

$$A_{i}^{*}=X_{i}B+\;\epsilon_{i}$$ (1)

where ***X***_***i***_ is a vector of independent variables that are hypothesized correlates of the accuracy of the *i*^th^ farmers’ beliefs; ***B*** denotes the estimated coefficients of those variables; and *ε*_*i*_ is a random error term. The term $A_{i}^{*}$ is an unobservable latent variable that denotes the accuracy of the *i*^th^ household’s belief where *A*_*i*_ = 0 if the household’s belief is accurate or $\left(A_{i}^{*}=0\right)$; and *A*_*i*_ = 1 if the household’s belief is inaccurate or $\left(A_{i}^{*}=1\right)$.

However, recognizing that the underlying decision processes that might explain correlates of a false positive may differ from those that explain correlates of a false negative, we introduce a multinomial logit to provide a more appropriate structure to the problem. Specifically, we consider three unordered outcomes denoted by (*j* = 0,1,2) to estimate an unobserved latent variable that describes the nature of the error in the household’s beliefs. These categories are defined as: *j* = 0 *if $A_{i}^{*}=0$*, denoting an accurate belief; *j* = 1 *if* $A_{i}^{*}=1$, denoting a Type I error; and *j* = 2 *if* $A_{i}^{*}=2$, denoting a Type II error.

We use data from a total of 380 household observations to estimate both the logit and multinomial logit models. Descriptive statistics are summarized in Table 8. Note that for the purposes of this immediate analysis, households committing Type II errors include both households that (1) believed they were cultivating non-Bt cotton but, according to the laboratory tests, were in fact, cultivating Bt cotton, and (2) farmers who did not know or did not respond to the question of whether they were cultivating Bt cotton. This definition varies from the explicit definition of a Type II error given earlier by making an arguably strong assumption that because adoption of a genetically modified variety is a conscious decision, in cases where farmers indicate that they are unaware of having made such a decision they are likely to hold the belief that they are not cultivating Bt cotton, thus committing a Type II error.

**Table 8:** Summary statistics for overall sample and selected sub-samples.

Variable	Description (unit)	Overall sample	Households with…		Households committing…	
			Accurate beliefs	Inaccurate beliefs	Type I errors	Type II errors^a^
		Mean (std. dev.)				
HH head age	Age (years)	46.26 (11.52)	46.03 (11.27)	46.76 (12.07)	46.61 (10.80)	46.84 (12.81)
*HH head education*						
No education	(1 = yes)	0.40 (0.49)	0.36 (0.48)	0.48 (0.50)	0.57 (0.50)	0.43 (0.50)
Primary or secondary education	(1 = yes)	0.53 (0.50)	0.54 (0.50)	0.50 (0.50)	0.41 (0.50)	0.55 (0.50)
Higher education	(1 = yes)	0.08 (0.27)	0.10 (0.30)	0.03 (0.16)	0.02 (0.15)	0.03 (0.16)
HH size	Household members (no.)	8.74 (4.20)	8.61 (4.36)	9.02 (3.85)	9.32 (3.59)	8.84 (4.00)
Experience with Bt	Years of Bt cultivation (years)	4.21 (1.71)	4.33 (1.67)	3.96 (1.78)	4.00 (1.46)	3.93 (1.95)
Contact with extension	Received cotton-related extension services (1/0)	0.27 (0.45)	0.31 (0.46)	0.20 (0.40)	0.16 (0.37)	0.22 (0.42)
Own land	Household cultivated own land/did not share-in or rent-in (1 = yes)	0.67 (0.47)	0.68 (0.47)	0.64 (0.48)	0.64 (0.49)	0.65 (0.48)
Distance to seed supplier	Time taken to reach supplier (minutes)	30.98 (35.71)	30.24 (31.65)	32.56 (43.23)	36.48 (48.37)	30.32 (40.16)
Total cultivated land area	Operated landholdings (acres)	4.24 (9.68)	4.64 (11.52)	3.38 (3.17)	4.01 (4.22)	3.02 (2.33)
Sealed and labeled package	(1 = yes)	0.67 (0.47)	0.68 (0.47)	0.61 (0.49)	0.71 (0.46)	0.56 (0.50)
*Agroclimatic zones*						
Northern Irrigated Plains	(1 = yes)	0.63 (0.48)	0.65 (0.48)	0.58 (0.50)	0.66 (0.48)	0.53 (0.50)
Sandy Dry Desert	(1 = yes)	0.12 (0.33)	0.14 (0.34)	0.10 (0.30)	0.16 (0.37)	0.07 (0.25)
Sulaiman Piedmont	(1 = yes)	0.04 (0.19)	0.03 (0.17)	0.06 (0.23)	0.02 (0.15)	0.08 (0.27)
Southern Irrigated Plains, Punjab	(1 = yes)	0.06 (0.24)	0.06 (0.23)	0.06 (0.23)	0.16 (0.37)	0 (0)
Southern Irrigated Plains, Sindh	(1 = yes)	0.11 (0.31)	0.09 (0.28)	0.16 (0.37)	0 (0)	0.25 (0.43)
Sand Dry Desert	(1 = yes)	0.04 (0.20)	0.04 (0.19)	0.05 (0.22)	0 (0)	0.08 (0.27)
Observations	(no.)	380	259	121	44	77

*Source*: Authors’ calculations.

Results from the logit and multinomial logit estimations are presented in Table 9, reported as marginal effects to illustrate how (a) for categorical variables, how much more likely the variable is to contribute to the outcome of interest (an inaccurate belief or commission of a Type I or Type II error); and (b) for continuous variables, how much more likely a unit change in the variable contributes to the outcome of interest. Results indicate that higher education is associated with reductions in the likelihood of inaccurate beliefs (in the logit estimation). School-level (primary or high school) education and higher education are also associated with a significant reduction in the likelihood of committing a Type I error (in the multinomial logit estimation). Results also suggest that contact with an agent is associated with reductions in the likelihood of inaccurate beliefs (in the logit estimation) and committing a Type I error (in the multinomial logit estimation).

**Table 9:** Estimated correlations between accuracy of beliefs about Bt cotton and strip test results.

Model	Model 1	Model 2		
	(Logit)	(Multinomial logit)		
Dependent variable	Accuracy of beliefs (1 = inaccurate)	Households committing…		
		No error	Type I errors	Type II errors
HH head age	0.00 (0.00)	0.00 (0.00)	-0.00 (0.00)	0.00 (0.00)
*HH head education*				
School education	-0.05 (0.06)	0.06 (0.06)	-0.12 (0.04)***	0.06 (0.04)
Higher education	-0.25 (0.07)***	0.27 (0.08)***	-0.17 (0.05)***	-0.10 (0.06)
HH size	0.01 (0.01)	-0.00 (0.00)	0.00 (0.00)	0.00 (0.01)
Experience with Bt	-0.02 (0.01)	0.02 (0.01)	-0.01 (0.01)	-0.01 (0.01)
Contact with extension	-0.08 (0.06)	0.07 (0.06)	-0.09 (0.04)**	0.03 (0.05)
Own land	0.03 (0.05)	-0.02 (0.05)	-0.03 (0.03)	0.05 (0.05)
Distance to seed supplier	0.00 (0.00)	-0.00 (0.00)	0.00 (0.00)	-0.00 (0.00)
Total cultivated land area	-0.01 (0.01)	0.01 (0.01)	0.00 (0.00)	-0.01 (0.01)
Sealed, labeled package	-0.01 (0.01)	0.02 (0.05)	0.03 (0.03)	-0.05 (0.04)
*Agroclimatic zones*				
Sandy Dry Desert	-0.06 (0.06)	0.06 (0.07)	0.01 (0.06)	-0.07 (0.05)
Sulaiman Piedmont	0.09 (0.12)	-0.11 (0.13)	-0.09 (0.06)	0.20 (0.13)
Southern Irrigated Plains, Punjab	0.04 (0.11)	-0.08 (0.10)	0.25 (0.10)**	-0.17 (0.02)***
Southern Irrigated Plains, Sindh	0.15 (0.09)	-0.21 (0.09)**	-0.14 (0.02)***	0.35 (0.09)***
Sand Dry Desert, Sindh	-0.00 (0.12)	-0.09 (0.13)	-0.14 (0.02)***	0.23 (0.13)*
Log likelihood	-225.94		-273.01	
Pseudo R squared	0.05		0.14	
LR chi squared	23.64		88.12	
No. of observations	380		380	

*Source*: Authors’ calculations.

*Notes*: Standard errors are in parentheses. Marginal effect (dy/dx) reported for factor levels is the discrete change from the base level. Coefficient estimates are significant at the

* 10 percent,

** 5 percent, and

*** 1 percent levels, respectively.

## Conclusion

This study explores the challenges in identifying the presence and effectiveness of the insect-resistance trait conferred by the Bt gene that was introgressed into cotton in Pakistan during the mid-2000s. Data from both a farmer survey and laboratory testing of plant tissue samples collected from the farmers’ main cotton plots confirm the widespread cultivation of the Bt cotton containing *cry1Ac* genes in Pakistan as well as the absence of *cry2Ab*. Yet there appears to be a significant disconnect between what farmers believe they are cultivating (Bt versus non-Bt cotton), what they are actually cultivating, and the extent to which the Bt gene is expressed at a level that is sufficient to defend against targeted pests. Across both provinces, 11 percent of farmers believed they were cultivating Bt cotton when, in fact, the Cry protein was not present in the tested tissue (i.e., a Type I error). Similarly, 5 percent of farmers believed they were cultivating non-Bt cotton when, in fact, the Cry protein was present in the tested tissue (i.e., a Type II error). In addition, 17 percent of all sampled farmers did not know if they were cultivating Bt cotton or not. Overall, 33 percent of farmers either did not know or were mistaken in their beliefs about the presence of the *cry* gene in the cotton they cultivated.

Results also indicate that the toxic protein expressed in the plant tissue samples collected from their fields fall below estimated threshold levels in a significant percentage of cases. As few as 10 percent of samples taken from farmers’ main plots meet the strict threshold of 1.5 μg/g, although 62 percent of samples meet the lower threshold at 0.6 μg/g. However, it is important to note that these estimated expression levels are also affected by factors related to tissue sample collection, handling, storage, and testing procedures. Despite this caveat, results suggest wide variability in both farmers’ beliefs and the gene expression, which may have implications for policy and regulation in Pakistan’s market for transgenic cotton seed.

The reasons for this disconnect may range from unintentional use of mixed (Bt and non-Bt) seed to intentional risk management strategies in which farmers plant multiple varieties on their farm or within a given plot. This raises questions about whether farmers experience yield or economic losses due to this disconnect and merits further research.

The study is not without caveats. First, challenges in obtaining, transporting, storing, and testing plant tissue samples from farmers’ fields can affect the accuracy of measurements of Bt gene expression levels. Second, because gene expression is known to vary substantially by genetic background, agroclimatic conditions, and agronomic practices that cannot be fully controlled for, it is difficult to make causal inferences between gene expression and Bt cotton performance.

## Supporting information

S1 Tables Household survey sampling frame. (PDF)

S1 Appendix Biophysical survey protocol and sampling frame. (PDF)

S2 Appendix Using ELISA results to validate strip test results. (PDF)
