# Supplementary material for: What are farmers really planting? Measuring the presence and effectiveness of Bt cotton in Pakistan
Source: PLoS One. 2017 May 4;12(5):e0176592. doi: 10.1371/journal.pone.0176592 (PMC5417514; doi:10.1371/journal.pone.0176592)
Supplement: S1 Tables — (PDF) [file pone.0176592.s001.pdf]

## S1 Tables: Household Survey Sampling Frame

### S1A Table—Sampling frame for 70 and 120 DAS, Punjab

| District | Village | No. of households | No. of households from which plant specimens were collected | |
|---|---|---|---|---|
| | | | *70 DAS* | *120 DAS* |
| Kasur | Nathoki | 14 | 14 | 14 |
| Kasur | Bharwal Kalan | 14 | 12* | 12* |
| Jhang | Kundal Khokhran | 14 | 12*,*** | 13* |
| Okara | Chak No. 045/3R | 14 | 14 | 14 |
| Okara | Chak 047/3R | 14 | 14 | 11** |
| Bahawalnagar | Momeka | 14 | 10*,*** | 13* |
| Bahawalnagar | Nathu Dacca | 14 | 13* | 13* |
| Bahawalnagar | Bidana Sharqi | 14 | 14 | 13** |
| Sahiwal | Chak No. 079-080/5-R | 14 | 14 | 14 |
| Sahiwal | Chak No. 118-12L | 14 | 13**** | 14 |
| Khanewal | Chak No. 007/8-AR | 14 | 13**** | 14 |
| Khanewal | Kot Bahadur | 14 | 11*,**** | 11** |
| Toba Tek Singh | Chak 303 JB/ Katohar Kalan | 14 | 8* | 7* |
| Toba Tek Singh | Chak 660/ 1 GB Itfaqabad | 14 | 14 | 14 |
| Multan | Chak No. 013/F | 14 | 14 | 14 |
| Multan | Gurdez Pur | 14 | 13* | 13* |
| Multan | Kot Rab Nawaz | 14 | 10* | 10* |
| Vehari | Chak No. 024/W.B | 14 | 14 | 14 |
| Vehari | Chak No. 163/W.B. | 14 | 13**** | 14 |
| Muzaffargarh | Sadiwahan | 14 | 13* | 13* |
| Muzaffargarh | Fatehpur Janubi 2 | 14 | 14 | 14 |
| Muzaffargarh | Bet Khan Wala | 14 | 13* | 13* |
| Lodhran | Chak No 388 W.B. | 14 | 11* | 11* |
| Lodhran | Khan Wah | 14 | 13**** | 14 |
| Bahawalpur | Ramzan Gulloo | 14 | 14 | 14 |
| Bahawalpur | Noru Arain | 14 | 9*,*** | 10* |
| Bahawalpur | Chak No. 003/ D N B | 14 | 14 | 13** |
| Rahimyar Khan | Chak No. 094/N. P. | 14 | 6* | 6* |
| Rahimyar Khan | Chak No. 147/P | 14 | 14 | 13** |
| Rahimyar Khan | Lal Shah | 14 | 13*** | 14 |
| Rajanpur | Wah Machka | 14 | 4* | 4* |
| Dera Ghazi Khan | Chak Qabool Shah | 14 | 13* | 13* |
| Dera Gazi Khan | Haji Kamand | 14 | 10* | 10* |
| Layyah | Kharal Azim Thal Jandi | 14 | 11* | 11* |
| Bhakkar | Panjgran Daggar | 14 | 11* | 10** |
| Faisalabad | Chak 656/GB | 14 | 10* | 5** |
| Faisalabad | Chak 112GB | 14 | 1* | 0** |
| Faisalabad | Chak 393/GB | 14 | 8*,*** | 7** |
| Faisalabad | Chak 550/ GB | 14 | 14 | 14 |
| Sargodha | Chak No. 149/150/N.B. | 14 | 5* | 5* |
| **Total** | | **560** | **461** | **456** |

Source: Authors

Notes: * Households dropped due to natural calamity; ** In addition to households that dropped at 70 DAS, some household plots could not be sampled at 120 DAS; *** Some households started sowing late so the samples could only be collected at 120 DAS; ****Those households are missing for which samples were misplaced.

**S1B Table—Sampling frame for 70 and 120 DAS, Sindh**

| District | Village | No. of households | No. of households from which plant specimens were collected | Location where diagnostic tests were conducted |
|---|---|---|---|---|
| Umer kot | Verasar | 14 | 14 | SAU* Tandojam |
| Badin | Khairpur | 14 | 14 | SAU Tandojam |
| Sanghar | 013-A-Jamrao | 14 | 12 | SAU Tandojam |
| Sanghar | Bhopi | 14 | 14 | SAU Tandojam |
| Khairpur | Nizamani | 14 | 5 | SALU** Khairpur |
| Ghotki | Atal Murdani | 14 | 9 | SALU Khairpur |
| Hyderabad | Pali Jani | 14 | 14 | SAU Tandojam |
| Nawabshah | Pat Piral | 14 | 11 | SAU Tandojam |
| Nawabshah | Sukhpur | 14 | 12 | SAU Tandojam |
| Nawabshah | Shahpur Jehania | 14 | 1*** | SAU Tandojam |
| Dadu | Hateen Digah | 14 | 12 | SALU Khairpur |
| Noushero Feroz | Kot Bahadur | 14 | 14 | SALU Khairpur |
| Total | | 168 | 132 | |

Source: Authors

Note: The sample frame remains consistent between 70 and 120 DAS.* Shah Abdul Latif University Khairpur; **Sindh Agricultural University, Tandojam;*** Cotton crop was destroyed in the other 13 cases.

**S1C Table—Number of tissues tested 2013**

| | | 70 DAS | | | | | 120 DAS | | | | | |
|---|---|---|---|---|---|---|---|---|---|---|---|---|
| **Province** | **Test/ tissue** | **Plant 1** | **Plant 2** | **Plant 3** | **Plant 4** | **Plant 5** | **Plant 1** | **Plant 2** | **Plant 3** | **Plant 4** | **Plant 5** | **All** |
| Punjab | Strip test/ | 461 | 461 | - | - | - | - | - | - | - | - | 922 |
| Sindh | leaf | 132 | 132 | 61 | 43 | 40 | 132 | 132 | 61 | 43 | 40 | 816 |
| Punjab | Strip test/ | 461 | 461 | - | - | - | 428 | 431 | - | - | - | 1,781 |
| Sindh | boll | 114 | 117 | 53 | 37 | 36 | 116 | 116 | 52 | 37 | 36 | 714 |
| Punjab | ELISA test/ | 461 | 461 | - | - | - | 457 | 457 | - | - | - | 1,836 |
| Sindh | leaf | 115 | 116 | 53 | 37 | 36 | 115 | 116 | 53 | 37 | 36 | 714 |
| Punjab | ELISA test/ | 461 | 461 | - | - | - | 428 | 431 | | | | 1,781 |
| Sindh | boll | 114 | 117 | 53 | 37 | 36 | 116 | 116 | 52 | 37 | 36 | 589 |
| **Total** | **All** | **2,319** | **2,326** | **220** | **154** | **148** | **1,792** | **1,799** | **218** | **154** | **148** | **9,153** |

Source: Authors
