# Supplementary material for: What are farmers really planting? Measuring the presence and effectiveness of Bt cotton in Pakistan
Source: PLoS One. 2017 May 4;12(5):e0176592. doi: 10.1371/journal.pone.0176592 (PMC5417514; doi:10.1371/journal.pone.0176592)
Supplement: S1 Appendix — (PDF) [file pone.0176592.s002.pdf]

S1 Appendix—Biophysical Survey Protocol and Sampling Frame

STEP 1: Activities undertaken prior to biophysical survey activities[1]

IFPRI/IDS provided to both UAF and NIGAB a document containing the information in Table S1 below to identify the mauza, farmer, and main plot that will be surveyed. Aside from this table, a particular code for each mauza and a drawing of main plot location were also provided to further identify the main plot as qualified by household respondent.

During the initial household survey conducted by IFPRI/IDS, the survey teams asked farmers for leftover seed (a minimum of 50 seeds) sown in their main plots, if available. These seeds were to be used to compare data collected from the field with data collected from plants grown under constant environmental conditions in a greenhouse. These seed and grow-outs were also to be used to conduct additional bioassays, if required.

The information in Table S1 below includes date of sowing, which enabled UAF and NIGAB to plan their field work at 70 and 120 days after sowing (DAS). NIGAB and UAF agreed to purchase the same brand of chemicals, biochemical, kits and reagents for use in the various laboratory techniques and procedures set forth in this study.

IFPRI/IDS sent one of its enumerators to accompany UAF and NIGAB survey technicians in the field to ensure proper identification of the district, mauza, farmer, and main plot from where samples should be collected. NIGAB and UAF deployed its survey technicians to collect leaf and boll samples as per the procedures described in STEP 2 and STEP 3.

Both NIGAB and UAF also provided IFPRI with plant tissue materials from the samples collected from the field site. These materials were used for independent, third-party testing.

[1] Based on the literature reviewed to date and key informant interviews, we assumed that only the Cry1Ac event (based on the Monsanto event MON 531) was prevalent in Pakistan at the time the tissue samples were collected. However, an additional diagnostic task were conducted in Step 1 to test for the presence and prevalence of Cry2Ab. The required work for this activity was for IFPRI enumerators to collect one sample of seed from each of the different Bt cotton varieties sold in local seed markets used by the survey respondents. ELISA tests were then be run on crushed seed samples for both Cry1Ac and Cry2Ab. This activity was to be organized and costed separately if resources are available. However, it was not required as the strip tests themselves could be used to test for the presence of Cry2Ab, as per the manufacturer’s specifications.

Table S1—Sample plot identification document

**Household Location/Address**		
**Code**		
A1	District	
A2	Tehsil	
A3	UC	
A4	Village	
A5	Address	
A6	Household number	
**Farmer's Information**		
A7	Name of respondent	
A8	Respondent's gender	
A9	Name of respondents' father	
A10	Mobile phone number where respondent can be reached	
A11	Name/location of the main cotton plot	
A12	Identification landmark for main plot (i.e. near canal)*	
A13	Distance of main plot to identification landmark *	Unit
A14	Estimated travel time from house to main plot (by normal transport)	Minutes
A15	Size of the main cotton plot *	Unit
A16	Name of the main cotton variety sown in the main plot	
A17	Date of sowing for this variety (mm ddyyyy)	
A18	When does respondent expect first picking. (mm ddyyyy)	
**Household GPS Information (in metric form)**		
A19	Elevation (m)	
A20	Latitude N	
A21	Longitude	
**Plot GPS Information (in metric form)**		
A22	Elevation (m)	
A23	Latitude N	
A24	Longitude	

STEP 2: Collection of leaf and boll samples at ~70 days after sowing (NIGAB/UAF)

The precise operating procedure for the biological sampling survey is as follows. *To guarantee that the results of NIGAB and UAF are strictly comparable, the field technicians and lab technicians received training on following uniform procedures.*

Before field sample collection:
Note that activities 1, 2, and 7 were conducted by UAF only. All other activities were conducted by NIGAB and UAF.

1. UAF (only) reared insect larvae of American bollworm (*Helicoverpa Armigera*) on artificial diet at 26±2°Cwith a relative humidity of 75 percent.
2. UAF (only) maintained laboratory cultures at the first instar to third instar stage for conducting bioassay at 70 DAS and 120 DAS.
3. NIGAB and UAF planned, scheduled, and implemented the collection of biological samples at 70 days after sowing using the information collected in Table S1−Question A17 so that samples were in fact collected at or around 70 DAS.
4. NIGAB and UAF purchased and prepared labels, polythene zip lock bags, plastic containers with perforated lids, artificial diet materials and plastic containers lined up with moist blotting paper for leaf sample collection, larvae collection and bolls collection respectively. Each label had detailed information of plot, sample number, and date.
5. NIGAB and UAF prepared a standardized training manual and arrange joint training for their field technicians who collected samples to guarantee uniform procedure of sample collection across and within teams. Training sessions for NIGAB and UAF teams were conducted on April 15th, 2013 at UAF.
6. NIGAB and UAF made arrangements to have adequate amounts of dried ice containers and/or cool packs and cool boxes for sample collection.

At each selected plot:

Activity 6 was conducted by UAF only. All other activities were conducted by NIGAB and UAF.

1. NIGAB and UAF field technicians tagged the plants selected for sample collection so the same plants could be easily identified for sample collection later at 120 DAS.

2. NIGAB and UAF field technicians collected two leaves *of similar size, color, and age* from the upper third part of each plant and combine them into one sample for testing. For leaf sample, technicians counted nodes from top to bottom and at the 4th node to 6th node, cut 2 leaves of the same size, age and color (lush green) per selected plant with scissors, took punches of two leaves together in pre-label eppendorf tube, and put the eppendorf tubes and remaining leaf in different pre-labeled zip lock bag. They placed a piece of same labeled paper in pre-labeled zip lock bag as well. The samples were stored in pre-labeled polythene zip lock bags and immediately placed in dry ice containers (in Punjab) and on cold gelpacks (in Sindh).

3. NIGAB and UAF field technicians collected 2 bolls from each plant (same plant, from where leaves were sampled), choosing bolls that measured 22mm, as verified by a ruler. These bolls were collected from the same plant from where the leaves were taken. Small hole were punched in the outer layer of each boll with the help of a puncher and kept in the pre-labeled eppendorf tube. The bolls with punched holes were placed in a pre- labeled zip lock bag and consider as one sample. They placed a piece of same labeled paper in pre-labeled zip lock bag as well. These boll samples were stored immediately in dry ice containers (in Punjab) or on cold gelpacks (in Sindh).

4. NIGAB and UAF field technicians repeated Procedures 1–3 for 2 different randomly selected plants in each selected plot. For the third to fifth selected plant in the field, leaf and boll samples were collected according to procedure outlined but instead of placing them in eppendorf tube, these samples were directly placed in the zip lock bags and stored in dry ice (in Punjab) and on cold gelpacks (in Sindh).

5. Note that several logistical problems were encountered in the field that limited the ability to adhere to a consistent procedure across Punjab and Sindh. In Punjab, the tissue samples were transported to UAF's laboratories under -20° C storage conditions using dry ice and maintained in a cold storage refrigerator at the same temperature for 7–15 days until tests were performed. In Sindh, the strip tests were performed on site immediately after collection, while the ELISA tests were performed within 24 hours of collection at one of two nearby university laboratories using the fresh tissue samples transported on cold gelpacks at 4–10° C.

6. NIGAB and UAF field technicians assessed the presence of *H. armigera, E. vitella* and *P. gossypiella* in each of the plants from where samples are collected. They logged numbers down in "Biophysical Survey Pro-forma (70 days after sowing)" detailed below. If found, NIGAB and UAF field technicians collected larvae of *H. armigera*, *E. vitella* and *P. gossypiella*in plastic containers with perforated lids and artificial diet materials. This was done for reporting purposes, not for bioassays.

7. For the bioassays, UAF (only) field technicians collected 5 bolls from each o*f two plant*s *of the* main field *of randomly* selected households of each mauza. UAF randomly selected two plots among the 14 households per mauza from IFPRI's HH sample. Three bolls and three larval instars were used for each bioassay. The remaining two bolls were kept at 4°C for back up.
    - The boll sizes were approximately 22 mm, as verified by a ruler. The field technicians collected the bolls of required size in a pre-labeled plastic container lined with moist blotting

paper to keep the bolls fresh. The bolls were collected preferably from the same plant where the other samples described above were taken for ELISA.

- While being transported to the lab, the bolls were placed in normal ice boxes or on cold gelpacks.
- Half of the bioassays (480) were performed on 70 DAS and the rest were carried out at 120 DAS. (The technique is outlined in detail separately below).

8. NIGAB and UAF field technicians collected information for each sample plot using the "Biophysical Survey Pro-forma (70 days after sowing)" detailed below.
9. NIGAB and UAF field technicians transported all collected samples to the lab. Samples were collected for proper storage and analysis. Samples were stored at -80°C until ELISA analysis. Samples for bioassay were stored at 4°C until used for bioassay within a day.

At the laboratory:[2]

The protocol indicated that both diagnostic tests were to be performed only on two of the five leaf samples and two of the five boll samples collected from each household's main cotton plot. The remaining three samples of both bolls and leaves were to be stored at -80°C for follow-up tests and third-party evaluation. This procedure was followed in Punjab.

In Sindh, however, strip tests were performed consecutively on each of the five tissue samples collected from the field until two positives were attained, and subsequently only positive results were subject to the ELISA test. In total, 9,153 diagnostic tests were conducted on the two types of plant tissue collected.

Apart from this variation, the laboratory procedures were conducted as follows.

1. Using a unique ELISA kit,[3] NIGAB and UAF conducted ELISA tests on leaf samples for Cry1Ac, and maintained the remaining samples for follow-up tests, as needed.
2. Using a unique ELISA kit, NIGAB and UAF conducted ELISA tests on two bolls samples per plot for Cry1Ac, and maintain the remaining samples for follow-up tests, as needed.
3. To enable comparison between results, NIGAB and UAF used the same ELISA kits and mathematical analysis of the ELISA readings. Slight variations in laboratory procedures are detailed below.
4. NIGAB and UAF used the same ground leaf samples to conduct immune strip tests that are designed to detect bothCry1Ac and Cry2Ab.

---

[2] Using their own financial and human resources, UAF will preserve part of the ground samples of leaves for further qualitative polymerase chain reaction (PCR). These ground samples will also be used for genomic DNA isolation using CTAB method. The isolated DNA will be used as template in pre optimized multiplex PCR, which will detect SadI genes (cotton endogenous gene), Cry1Ac and Cry2Ab genes and Mon531 event in the collected samples by using the following primers. Multiplexing makes the technique economic, cost effective and more reliable. This study component will complement the Bt expression data analysis and rule out false positive results. This component will be carried out and covered by UAF.

[3] The suggested ELISA kit will be the EnvirologikQuantiPlate Kit for Cry1Ab.

STEP 3: Collect leaf and boll samples at 120 days after sowing (NIGAB/UAF)

Before field sample collection:

Activity 1 was conducted by UAF only. All other activities were conducted by NIGAB and UAF.

1. UAF (only) maintained larval cultures of *H. armigera* for the remaining bioassays.
2. NIGAB and UAF planned, scheduled, and implemented the collection of biological samples at 120 days after sowing using the information collected in Table S1−Question A17 so that samples were in fact collected at or around 120 DAS.
3. NIGAB and UAF purchased and prepared labels, polythene zip lock bags, plastic containers with perforated lids, artificial diet materials, and plastic containers lined with moist blotting paper for leaf sample collection, larvae collection and bolls collection respectively. Each label had detailed information of plot id, sample number, and date.
4. NIGAB and UAF made arrangements to have adequate amounts of dried ice containers and/or cool boxes for sample collection.

At each sample plot:

Activity 6 was conducted by UAF only. All other activities were conducted by NIGAB and UAF.

1. NIGAB and UAF field technicians located plants tagged and selected for sample collection at 70 DAS and used these same plants for tissue collection.
2. NIGAB and UAF field technicians collected two leaves *of similar size, color, and age* from the upper third part of each plant (the same plant from where leaves were sampled at 70 DAS) and combined them into one sample for testing. For leaf sample, the field technicians counted nodes from top to bottom at 4th node to 6th node, cut 2 leaves of same size, age and color (lush green) per selected plant with scissors, took punches of two leaves together in pre-labeled eppendorf tube, and put the eppendorf tubes and remaining leaf in different pre-labeled zip lock bag. They placed a piece of the same labeled paper in pre-labeled zip lock bag as well. The samples were stored in pre-labeled polythene zip lock bags and immediately placed in dry ice containers (in Punjab) and on cold gelpacks (in Sindh).
3. NIGAB and UAF field technicians collected 2 bolls from each plant (the same plant from where leaves were sampled at 70 DAS), choosing bolls that measure 22mm, as verified by a ruler. These bolls were collected from the same plant from where the leaves were taken. Small holes were punched in the outer layer of each boll with the help of puncher, and these punches were placed in the pre-labeled eppendorf tube. These bolls with punched holes were placed in a pre- labeled zip lock bag and consider as one sample. They placed a piece of the same labeled paper in pre-labeled zip lock bag as well. These boll samples were stored immediately in dry ice containers (in Punjab) and on cold gelpacks (in Sindh).
4. NIGAB and UAF field technicians repeated Procedures 1–3 for 2 different randomly selected plants in each selected plot. For the third to fifth selected plant in the field, leaf and boll samples were collected according to procedure outlined but instead of placing them in eppendorf tube these samples were directly placed in the zip lock bags and stored in dry ice.

5. NIGAB and UAF field technicians assessed the presence of *H. armigera, E. vitella* and *P. gossypiella* in each of the plant from where samples are collected. They logged numbers down in “Biophysical Survey Pro-forma (70 days after sowing)” detailed below.

6. For the bioassays, UAF (only) field technicians collected 5 bolls from each o*f two plant*s *of the main field of randomly* selected households of each mauza. UAF randomly selected 2 plots among the 14 households per mauza from IFPRI’s household sample. Three bolls and three larval instars were used for each bioassay. The remaining two bolls were kept at 4°C for back up.

    - The boll size were approximately 22 mm, as verified by a ruler. The field technicians collected the bolls of required size in a pre-labeled plastic container lined with moist blotting paper to keep the bolls fresh. The bolls were to be collected preferably from the same plant where the other samples described above were taken for ELISA.

    - While being transported to the lab, the bolls were placed in normal ice boxes or on cold gelpacks.

7. NIGAB and UAF field technicians collected information for each sample plot using the “Biophysical Survey Pro-forma (120 days after sowing)” detailed below.

8. NIGAB and UAF field technicians transported all collected samples to the lab. Samples are collected for proper storage and analysis. Sample will be stored at -80° C until ELISA analysis. Samples for bioassay were stored at 4°C until used for bioassay within a day.

At the laboratory:[4]

Activities 1-5 were conducted by NIGAB and UAF.

1. NIGAB and UAF used two samples of leaf and two samples of bolls (collected in eppendorf tubes) in a given field to conduct ELISA tests. The remaining three samples of leaf and bolls (in the zip lock bags) were stored at -80°C for follow-up tests and independent third-party validation, as needed. Note that procedures varied between NIGAB and UAF here.
2. Using a unique ELISA kit,[5] NIGAB and UAF conducted ELISA tests on two leaf samples per plot for Cry1Ac, and maintained the remaining samples for follow-up tests, as needed.
3. Using a unique ELISA kit, NIGAB and UAF conducted ELISA tests on two bolls samples per plot for Cry1Ac, and maintained the remaining samples for follow-up tests, as needed.
4. To enable comparison between results, NIGAB and UAF used the same ELISA kits and mathematical analysis of the ELISA readings.
5. NIGAB and UAF used the same ground leaf samples to conduct immunostrip tests that detect both Cry1Ac and Cry2Ab.

ELISA protocols and procedures:

The ELISA analysis was performed for the leaf and boll samples collected from the field at 70DAS and 120DAS using following protocol.

---

[4] Procedure detailed in footnote 2 for 70 DAS were repeated by UAF at 120 DAS only if samples collected at 120 days for ELISA cannot be verified to be from the same plant as the samples collected at 70 days

[5] The suggested ELISA kit were Envirologik QuantiPlate Kit for Cry1Ab.

1. Preparation of Solutions. The solutions for the assay were prepared as follows.

a. Wash buffer: The contents of wash buffer salt (phosphate buffered saline, pH 7.4 – Tween® 20) were added to one liter of distilled or deionized water and stirred to dissolve.

b. 1x Extraction/Dilution Buffer (for leaf tissue sampling): The entire contents of the bottle of 5X Extraction/Dilution Buffer (35 mL) supplied in the kit was added to 140 mL of distilled or deionized water in a suitable container and was mixed thoroughly to dissolve.

2. Sample Extraction: Two leaf punch samples (approximately 10 milligrams each) were taken by snapping the cap of disposable eppendorf tubes down on the leaf. Note that there were differences in the way the samples were subsequently weighed for testing. In Punjab, the actual weight was measured by analytical balance for each tissue sample, while in Sindh a constant sample weight of 20 mg was used in calculations.

Next, the tissue was ground by rotating the pestle against the sides of the tube with twisting motions. This process was continued for 20-30 seconds or until the leaf tissue was ground well.

Next, the extraction/dilution buffer was added to the tube. 0.5 mL of 1X Extraction/Dilution Buffer was added to the tube. Grinding step was repeated to mix tissue with 1X Extraction/Dilution Buffer. This protocol was repeated for each sample to be tested and a new tube and pestle was used for each. The solids were allowed to settle in each tube for a few minutes.

3. Sample Dilution: There were also minor procedural differences between testing in Punjab and Sindh, such as the choice of dilution factor. In Punjab, the sample extracts were diluted to 1:11 prior to assay; in Sindh, the sample extracts were diluted at 1:9. Next, 0.5 mL 1X Extraction/Dilution Buffer was added to dilution tubes labeled for each sample, and 50 µL of the sample extract was add and mixed.

4 Running of Assay: The assays were performed as follows.

a. i. Negative controls, calibrators, and sample extracts (100 µL of each) were added to their respective wells of 96-well plate. We followed this same order of addition for all reagents.

b. Thorough mixing of the contents of the wells was done by moving the strip holder in a rapid circular motion on the bench top for a full 20-30 seconds. Mixing was done carefully to avoid cross-contamination.

c. The wells were covered with a tape or Parafilm to prevent evaporation and incubated at ambient temperature for 15 minutes. An orbital plate shaker was used to shake the well contents at 200 rpm.

d. An Enzyme Conjugate (100 µL) was added to each well.

e. The contents of the wells were thoroughly mixed as described above.

f. The wells were covered with new tape or Parafilm and incubated at ambient temperature for one hour. An orbital plate shaker was used to shake the well contents at 200 rpm.

g. After incubation, the covering was removed carefully and the contents of the wells were emptied vigorously into a sink. The wells were completely flooded with Wash Buffer and then shook till empty. The wash step was repeated three times. The inverted plate was slapped several times on a paper towel to remove as much water as possible.

h. The substrate (100 µL) was added to each well.

i. The contents of the wells were thoroughly mixed and covered with new tape or Para film and incubated for 30 minutes at ambient temperature.
