# Supplementary material for: What are farmers really planting? Measuring the presence and effectiveness of Bt cotton in Pakistan
Source: PLoS One. 2017 May 4;12(5):e0176592. doi: 10.1371/journal.pone.0176592 (PMC5417514; doi:10.1371/journal.pone.0176592)
Supplement: S2 Appendix — (PDF) [file pone.0176592.s003.pdf]

S2 Appendix: Using ELISA Results to Validate Strip Test Results

To validate the findings described in Section 4.2, we compare strip test results with ELISA results. We assume that a positive strip test is validated by ELISA results when the latter detect the Bt protein at some level greater than zero. Table S2A below describes the classification criteria used to determine whether a tissue sample from Punjab or Sindh was categorized at Bt, non-Bt, or inconclusive based on the combination of data from strip test and ELISA results.

These results allow for the construction of an alternative classification of varieties as Bt or non-Bt that uses a combination of the strip test and the ELISA test results. To construct this classification, we first establish a lower bound for Bt protein expression that accounts for values set by the bioassays. These values fall between 0.598 µg/g and 1.590 µg/g for $LD_{50,}$ $LD_{95,}$ respectively. Second, we establish a conservative although subjective lower bound at 0.09 µg/g for "ineffective" protein expression, as it is unlikely that the targeted insect population can be effectively controlled at such lower levels.

Third, we determine that a farmer is planting Bt cotton if (1) all leaves collected from his main cotton plot at 70 DAS tested positive for *Cry1Ac* based on the strip test, and (2) the ELISA test results are greater than 0.09 ug/g. Similarly, we determine that a farmer is not planting Bt cotton if (1) all leaves collected from his main plot tested negative for *Cry1Ac* based on the strip tests, and (2) ELISA test results are less than 0.09 ug/g. Finally, anything that falls between these two strict classifications are reported as "inconclusive." Using the criteria specified in Table S2A below for the classification of Bt, non-Bt or inconclusive, the results for Punjab and Sindh are summarized in Table S2B below.

The results indicate that 50 percent of plots tested were planted with "effective" Bt cotton, and just 7 percent were planted with non-Bt cotton. This suggests that adoption rates based on strip test results alone may over-estimate adoption by more than 30 percentage points, while adoption rates based on farmer self-reporting may over-estimate adoption by 35 percentage points.

Next, we return to the question of farmers' beliefs about whether they were planting Bt cotton (Table S2C below). We do this by revising lower threshold for lethal concentration at 0.589 ug/g and apply it to results from ELISA results from leaf tissue samples taken at 70 DAS. First, findings indicate that 52 percent of the cases in which farmers believed that they *did* plant Bt cotton were consistent with the detailed classification indicating the presence of effective Bt gene expression. Type 1 errors occurred in just 3 percent of cases where farmers reported that they had planted Bt cotton, while 46 percent of these cases were inconclusive. Second, 7 percent of the cases in which farmers believed that they *did not* plant Bt cotton were consistent with the detailed classification indicating the presence of effective Bt gene expression. Type 2 errors occurred in 43 percent of cases where farmers reported that they had not planted Bt cotton, while 51 percent were inconclusive. Across the entire sample, Type 1 and Type 2 errors accounted for 9 percent of all cases.

S2A Table—Detailed classification criteria based on strip and ELISA test results and Bt protein expressions below 0.09 ug/g

Classification	Province	Strip test result					ELISA results (ug/g)				
		Plant 1	Plant 2	Plant 3	Plant 4	Plant 5	Plant 1	Plant 2	Plant 3	Plant 4	Plant 5
Bt	Punjab	Positive	Positive	-	-	-	≥ 0.09	≥ 0.09	-	-	-
	Sindh	Positive	Positive	-	-	-	≥ 0.09	≥ 0.09	-	-	-
Non-Bt	Punjab	Negative	Negative	-	-	-	< 0.09	< 0.09	-	-	-
	Sindh	Negative	Negative	Negative	Negative	Negative	-	-	-	-	-
Inconclusive	Punjab	Any negative strip test result		-	-	-	Any ELISA result <0.09		-	-	-
	Sindh	Any negative strip test result					Any ELISA result <0.09				

Source: Authors.
Note: "-" denotes that the specified test was not performed.

Table S2B provides a cross-tabulation of these findings, indicating that strip test results are generally supported by ELISA results, although detectable levels of Bt expression are found in a number of tissue samples that returned negative strip tests. This cross-tabulation also places a large share of observations (43 percent) into the "inconclusive" category.

S2B Table—Combined ELISA and strip test results

Combined test results	Punjab		Sindh		All	
	#	%	#	%	#	%
Bt						
-All positive strip tests and ELISA results at ug/g ≥ 0.09	229	49	71	54	300	50
Non-Bt						
-All negative strip tests and ELISA results at ug/g < 0.09	9	2	32	24	41	7
Inconclusive						
-All other results	226	49	29	22	255	43
Total	464	100	132	100	596	100

Source: Authors.

S2C Table—Comparison of farmers' perceptions and detailed classification of Bt cotton leaf tissue samples collected at 70 DAS indicating the presence of effective Bt protein expression

	Combined test results (Strip, Elisa, and Bioassay 0.58 ug/g threshold)							
	Bt		Non-Bt		Inconclusive		Total	
	#	%	#	%	#	%	#	%
**Bt**	226	51.8	11	2.5	199	45.6	436	100.0
**Non-Bt**	43	42.6	7	6.9	51	50.5	101	100.0
**Don't know**	3	100.0	0	0.0	0	0.0	3	100.0
**No response**	28	52.8	1	1.9	24	45.3	53	100.0
**Total**	300	50.6	19	3.2	274	46.2	593	100.0

Source: Authors
